# Estimates of the Lung Cancer Cases Attributable to Radon in Municipalities of Two Apulia Provinces (Italy) and Assessment of Main Exposure Determinants

**DOI:** 10.3390/ijerph15061294

**Published:** 2018-06-20

**Authors:** Giovanni Maria Ferri, Graziana Intranuovo, Domenica Cavone, Vincenzo Corrado, Francesco Birtolo, Paolo Tricase, Raffaele Fuso, Valeria Vilardi, Marilena Sumerano, Nicola L’abbate, Luigi Vimercati

**Affiliations:** Unit of Occupational Medicine, Regional University Hospital “Policlinico-Giovanni XXIII”, Section “B. Ramazzini”, Interdisciplinary Department of Medicine, University of Bari, Piazza G, Cesare, 11, 70124 Bari, Italy; grazianaintranuovo@gmail.com (G.I.); domenica.cavone@uniba.it (D.C.); corrado.vincenzo@uniba.it (V.C.); francesco.birtolo85@gmail.com (F.B.); paotric1990@hotmail.it (P.T.); raffaele9275@live.it (R.F.); valescratta@hotmail.it (V.V.); sumeranomarilena@libero.it (M.S.); nicolalabbate@unifg.it (N.L.); luigi.vimercati@uniba.it (L.V.)

**Keywords:** radon exposure, WLM (working level month), lung cancer, constructions typology, annual risk estimation, precaution principle

## Abstract

Indoor radon exposure is responsible for increased incidence of lung cancer in communities. Building construction characteristics, materials, and environmental determinants are associated with increased radon concentration at specific sites. In this study, routine data related to radon measurements available from the Apulia (Italy) Regional Environmental Protection Agency (ARPA) were combined with building and ground characteristics data. An algorithm was created based on the experience of miners and it was able to produce estimates of lung cancer cases attributable to radon in different municipalities with the combined data. In the province of Lecce, the sites with a higher risk of lung cancer are Campi Salentina and Minervino, with 1.18 WLM (working level months) and 1.38 WLM, respectively, corresponding to lung cancer incidence rates of 3.34 and 3.89 per 10 × 10^3^ inhabitants. The sites in the province of Bari with higher risks of lung cancer are Gravina di Puglia and Locorotondo, measuring 1.89 WLM and 1.22 WLM, respectively, which correspond to an incidence rate of lung cancer of 5.36 and 3.44 per 10 × 10^3^ inhabitants. The main determinants of radon exposure are whether the buildings were built between 1999 and 2001, were one-room buildings with porous masonry, and were built on soil consisting of pelvis, clayey sand, gravel and conglomerates, calcarenites, and permeable lithotypes.

## 1. Introduction

Globally, products of radon decay are responsible for approximately 50% of the effective dose that every person receives in exposure to natural sources of radiation. Radon is a naturally occurring radioactive noble gas. Colorless and odorless, it is naturally present in soils, rocks and building materials, from where it spreads with relative ease, mixing with other gases present in the atmosphere and reaching typical concentrations of 10 Bq/m^3^. However, these values typically present in the atmosphere are not worrisome for general health. By contrast, the situations in which this gas accumulates in closed environments (indoor radon) are different. In these situations, in fact, the concentrations can reach values even higher than 1000 Bq/m^3^, and the repercussions on health areimportant; radon can insidiously penetrate into living neighborhoods and through the slots of the building envelope (non-hermetic points), thus accumulating in the air inside the premises [1].

In Apulia, the threshold values (annual average concentrations) of radon gas were equal to 300 Bq/m^3^, in line with the European directive (2013/59/EURATOM [2]) and National Radon Plan [3].

There are three radon isotopes that are a result of the decay of elements ^238^U, ^232^Th, and ^235^U. Those isotopes originate from ^222^Rn, ^220^Rn (also called actinon), and ^219^Rn (also known as thoron), respectively. Although ^220^Rn and ^219^Rn are irrelevant, determinable by their short decay times, ^222^Rn derived from radioactive decay in the uranium decay chain is more important from a radioactive point of view. Radon (^222^Rn) has a half-life of 3.82 days and forms radioactive lead, bismuth, and polonium “daughters” whose emitted α-radiation is dangerous [4]. It does not create chemical bonds and tends to migrate within the original material, moving in relation to the concentration, pressure and temperature gradients. It is also a heavy gas (with a density equal to 0.75 times that of air) and moderately soluble in water, therefore representing an important vehicle for the transport of radon even at great distances from the place of formation. Thus, radon easily escapes from water—for example, by making air bubbles or simply shaking it [5].

Radon expulsion from the crystal lattice of the mineral is responsible for the high concentrations of radon in soil and subsoil gases. In particular, in the decay of ^226^Rn, an α particle is emitted, and the newly formed radon atom undergoes the so-called “recoil effect” (0.02–0.07 μm) in the opposite direction. The position of the radon atom in the grain and the recoil direction of the atom itself can determine whether radon will leak from the crystal lattice of the mineral that generated it [6].

Under these conditions, three different situations can occur: the radon atom remains in the granule; the radon atom penetrates a nearby granule; or the crystal lattice expels the radon atom and is subsequently removed from soil gases or water. Only in the third case, is radon free to move through the ground, reaching the surface and finally spreading into the atmosphere. There is an association between the permeability of the soil and radon mobility. A gravelly or craggy soil will allow radon gas to move easily through the rocky layers, while clayey and possibly humid horizons will present a certain resistance to its passage. Furthermore, the action of some gases, such as carbon dioxide, help radon migrate to the surface or water, where it is soluble [7,8].

The main health effect of decaying radon products is an increased risk of lung cancer [9]. Regarding the evidence for this carcinogenic effect, since 1988, in the classification of carcinogens carried out by the IARC (International Agency for Research on Cancer) on behalf of the WHO (World Health Organization), the decay products of radon are classified in group 1 [10]. A series of case-control epidemiological studies conducted on individuals exposed in homes in North America, Europe, and Republic of China confirmed the carcinogenic effect [11].

Radon exposure is the second most common cause of lung cancer after smoking; in particular, some authors have shown that the attributable risks (ARs) of lung cancer deaths due to indoor radon exposure were higher among never-smokers than ever-smokers, while most of the lung cancer deaths attributable to radon occurred among ever-smokers [12,13]. Lung cancer deaths induced by radon were slightly higher among females than males, while after stratifying populations according to smoking status, the percent of ARs was similar between genders.

Miners occupationally exposed to high concentrations of radon showed a statistically significantly high risk of lung cancer [14].

Low and average concentrations of radon, rather than a high concentration, can cause most radon-induced lung cancers. In general, exposure to a high concentration of radon in closed environments is rare. The “stochastic” effects of radon exposure are closer to the “precautionary principle”.

The aims of this study were as follows: to describe the radon concentration in the municipalities of the Apulia provinces of Bari and Lecce; to study the distribution of the radon levels above the 75th percentile in those municipalities; to estimate the annual risk of developing lung cancer attributable to domestic exposure to indoor radon and to assess the associations between geo-urbanistic variables and radon measurements above the 75th percentile.

## 2. Material and Methods

The measurements of the radon gas concentration (annual average), based on techniques prescribed by the regional legislation and adopted by the Apulian Regional Environmental Protection Agency (ARPA), require the use of passive instrumentation, using passive dosimeters of the CR-39 type that respond to nuclear tracks. For each monitored environment, based on the duration of effective monitoring, the minimum detectable concentration was calculated considering the minimum detectable exposure value indicated by the supplier of the dosimeters (CR-39), supplied by Tecnorad (Verona, Italy), and was provisionally set at a value of 25 kBqh/m³ divided by the actual duration of the measure. For analysis of the dosimeters, the chemical development was performed using the Politrack automated optical measuring system, which was developed by the Mi.am SRL company (Piacenza, Italy). This technique is the most suitable for large-scale monitoring and allows for the storage of the measured value information over time, making it available for repeated and subsequent readings [15].

### 2.1. Radon Concentration Measurements in the Bari Province

The data were measured for 31 municipalities in the years 2004–2005 for two consecutive semesters (autumn–winter and spring–summer) during a regional radon survey (ARPA). The monitoring of workplaces and, in particular rooms located on the ground and in the basement or basement floors was carried out; these areas are the principal areas where the greatest concentrations of radon gas are recorded. Data on the minimum, maximum, and average concentrations of the various radon values obtained by dosimeters were used. Furthermore, for each municipality investigated, ARPA, through a questionnaire, collected data related to the building characteristics, number of examined rooms, and number of adopted dosimeters.

### 2.2. Radon Concentration Measurements in Lecce Province

The data were collected during a radon survey of 20 municipalities carried out from 2013 to 2015. Radon measurements were carried out exclusively in buildings for residential use (100% of the sample) with a prevalence of measurements carried out on the ground floor. The examined houses are approximately 20 for each municipality, including 10 houses located in the historic center and built before 1950.

For the municipalities of both provinces, no reliable data on the ventilation of the premises monitored were available due to the use of current data collected for routine ARPA measurements.

### 2.3. Concentrations of Radon Inside the Houses

The concentrations are closely associated with structural and use factors; the considered variables are as follows: year of building construction; number of rooms; construction material; and building plan. The National Institute of Statistics provided these data that had been collected during the survey of the 15th general census of the population and housing of the year 2011 [16].

**Figure 1:**
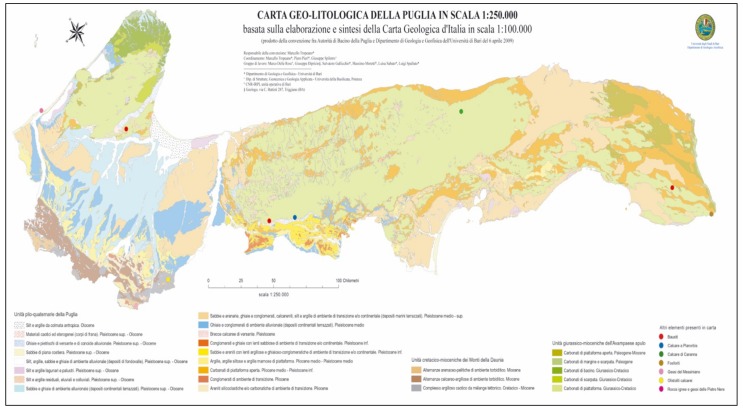
Geo-lithological map of Puglia, scale 1:250.000. Apulia in scale 1:250000 (produced by the agreement between the Apulia Basin Authority and the Department of Geology and Geophysics of the University of Bari on 6 April 2009).

The concentration data were categorized on the 75th percentile value of the cumulative frequency distribution of the concentrations measured in the 51 municipalities studied (145 Bq/m^3^). The environment of the measured sites is characterized by the following three geological and lithological variables: soil type, age of soil formation, and presence of faults or tectonic plates. The relative database has been recovered from the geo-lithological map of the Higher Institute for Environmental Protection and Research [17] (Figure 1).

### 2.4. Four Types of Soil

Four types of soil were identified and distinguished that were different in morphology, composition and age of formation in the Apulian region. The classification includes the following: soil no. 1 (composition = pebbles, clayey sands, gravels or conglomerates, calcarenites, permeable lithotypes; age = middle pliocene−lower pleistocene); soil no. 2 (composition = limestone, dolomitic limestone and dolomite, locally lower bauxitic levels; age = upper cretaceous, locally from the lower cretaceous or up to the palogene); soil no. 3 (composition = organogenic and detrital limestone; locally sandstone; age = miocene−pleistocene lower than places from the oligocene); soil no. 4 (composition = limestone, dolomitic limestone, and dolomite locally with poorly permeable marly levels made of clay, age = lower jurassic−cretacic). The characterization of the building associated with the different soil types was carried out by means of a simple codification (1 = presence; 0 = absence).

### 2.5. Different Structural Characteristics

The different structural characteristics of the variables related to the radon concentration inside the houses were as follows: epoch of building construction (before 1919, 1919–1945, 1946–1961, 1962–1971, 1972–1981, 1982–1991, after 1991, 2001–2005, after 2006); number of floors above the ground level; construction materials (load-bearing, concrete, other); number of rooms (1, 2, 3, or 4, from 5 to 8, from 9 to 15, >16). These data were provided by ISTAT of Bari and concerned the survey of the 15th census in 2011 [16].

### 2.6. Estimate of the Annual Risk of Lung Cancer Attributable to Radon

**Figure 2:**
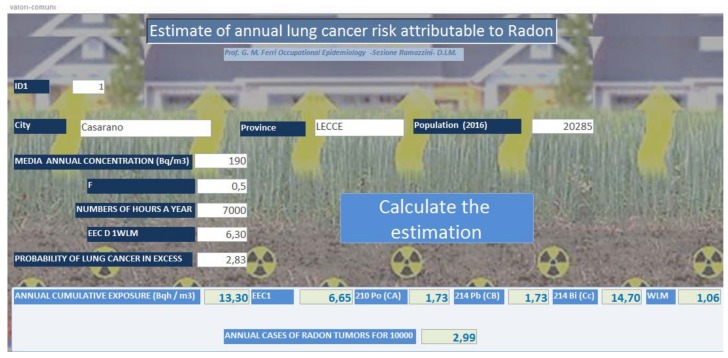
Mask of the software realized to produce the estimates of annual lung cancer attributable to radon. F (equilibrium factor) is the ratio between the concentration of the descendants and that of the radon; F (equilibrium factor) = EEC/C (EEC is the radon concentration equivalent to equilibrium); C is the radon concentration; EEC = 0.10CA + 0.52CB + 0.38Cc (CA, CB, Cc represent respectively the concentration of activity of the ^210^Po, ^214^Pb, ^214^Bi radon decay products); WLM (working level month) is the cumulative exposure of 1 WL (working level) per working month of 170 h is the conventional exposure unit.

The estimates were realized using software realized by our unit. The realization of the software was finalized to have a flexible tool, to be easy to use, and to be based on the data set for each municipality. The software calculated the expected annual tumor cases for radon at 10,000. It is extremely intuitive. It was developed in Microsoft Access 2010 (Microsoft Corporation Redmond, Redmond, WA, USA) and works with Microsoft Windows operating systems, versions XP and later. A table was created in Access with the calculated fields related to the following: (1) population of municipality; (2) mean annual concentration (Bq/m^3^); (3) F (equilibrium factor); (4) number of hours a year; (5) EEC (equilibrium equivalent radon concentration) 1 WLM (working level months); (6) probability of lung cancer in excess. A management mask was set up for the insertion/modification/searching of data related to the reference municipality. Once the data entry was complete, the “Calculate the Estimation” button was clicked to produce the estimate. The data produced by the software were as follows: (1) annual cumulative (Bq/m^3^); (2) EEC1; (3) ^210^Po(Ca); (4) ^214^Pb(CB); (5) ^214^Bi(Cc); (6) WLM; and (7) estimates of the annual number of lung cancer cases attributable to radon (Figure 2).

The model used to determine these lung cancer estimates, caused by the inhalation of radon and its descendants such as ^210^Po, ^214^Pb, and ^214^Bi was derived from the model used in the cohort study on miners working in the underground urinals, performed by the University of New Mexico “Health Sciences Center” [12] and used in Italy in a previous study [18]. The method was based on the study of the working level month (WLM), which represents the conventional unit of exposure to radon evaluated over the entire life of mining workers. The WLM corresponds to the cumulative exposure of 1 WL for a work month of 170 h; therefore, the probability of lung cancer, at the exposure of 1 WLM from 18 to 64 years, was estimated to be 2.83 × 10^−4^. The calculation refers to an equilibrium factor F = EEC × C^−1^. The EEC = 0.10CA + 0.52CB + 0.38Cc (CA, CB, and Cc respectively represent the activity concentrations of ^210^Po, ^214^Pb, and ^214^Bi, which are radon decay products) and C the cumulative mean annual exposure [19]. In this regard, an equilibrium factor F is introduced, defined as the ratio between the EEC in the atmosphere under examination and the true concentration of radon in the atmosphere; this real concentration of radon in the atmosphere is given by the ratio between the concentration of the descendants and that of radon, the percentage of the descendants that attaches themselves to the particulate, the size of the aerosols, and the breathing rate of people. Typical F values for homes range from 0.3 to 0.7 [20].

The different uses (workplaces or residential use) of radon-monitored buildings in the two provinces (Bari and Lecce) were not crucial for the aims of our study. Both building types were, in fact, associated with the same determinants (geological underground characteristics, number of rooms, number of floors, wall types and years of construction). Additionally, the working level month, important for the assessment of the number of lung cancer cases attributable to radon, was based on the experience of the workplace of miners [19].

### 2.7. Statistical Analysis

After gathering all the data on the different variables (type of subsoil, type of masonry, construction time, number of interiors and number of grounds) of the various municipalities in a single overall database using Excel (Microsoft Corporation Redmond, Washington, DC, USA) these data were transformed in a database compatible with the statistical software Stata 12 (Stata Corp. LLC, College Station, TX, USA). Using this software, univariate and multivariate analyses were performed using a non-conditional logistic regression model.

Therefore, no simple relationship exists between the concentration of radon in air and the environment and the dose absorbed by a person living in that environment. However, measurements of the radon concentration can serve as a reference for the risk assessment. Moreover, these measures are used to study how the characteristics of the dwellings (type of construction, soil geology, ventilation of rooms) affect the accumulation of radon in the rooms.

## 3. Results

### 3.1. Basic Description of the Variables

**Figure 3:**
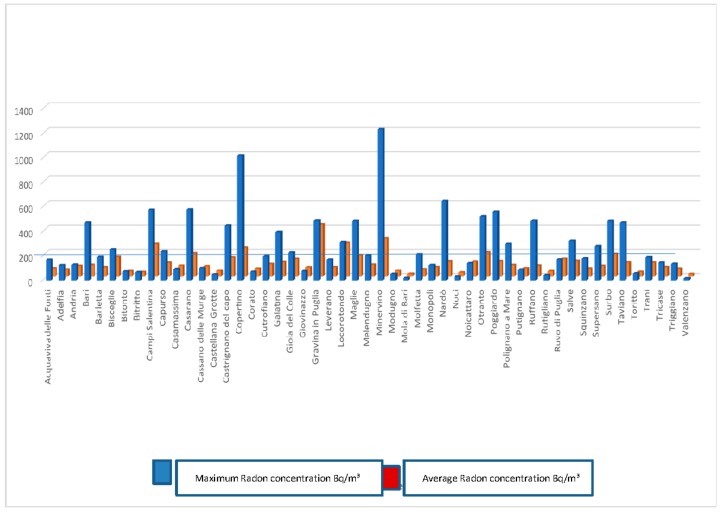
Radon concentrations in the 51 studied municipalities.

The distribution of average annual concentrations (Bq/m^3^) in the Apulian region show values ranging between 500 and 800 Bq/m^3^ near Lecce and Leuca and between 300 and 400 Bq/m^3^ in Valle d’Itria. The annual maximum and average concentration data related to 31 municipalities in the province of Bari and 20 municipalities in the province of Lecce are reported (Figure 3).

**Figure 4:**
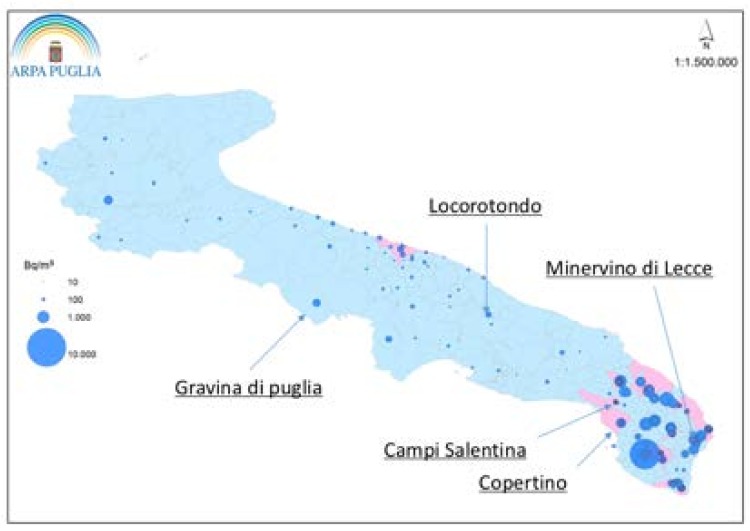
Annual Average concentration of Radon in Apulia region. Notes: In the map, blue circles represent the housing, schools, and workplaces in which ARPA Puglia made the measurements of the concentration of radon. The diameter of each circle is proportional to the average annual concentration measured. The municipalities for which data from at least seven in-house measurements are available and are highlighted in pink. This agency highlights on the basis of the experience gained in the field and especially of what was reported in the literature, that the only safe method to determine the concentration of radon gas inside a closed environment, independently of the results of the campaigns of previously measurements carried out in the same region, same province, the same municipality, even as an adjoining palace, is the direct measure. It was shown that adjacent buildings, with identical constructive characteristics, can have different radon concentrations. Therefore, to evaluate the concentration of radon in a closed environment, starting from experimental measurements carried out in other closed environments in other buildings that exist in the same area, municipality, or province (such as the data shown in the map) could lead to gross approximations for both defect and excess (Modified by ARPA publications, 2013).

Among the municipalities of the Bari province, the mean radon concentration found in these 31 municipalities was between 18 and 427 Bq/m^3^, the highest average concentration was found in Gravina di Puglia (427 Bq/m^3^) and Locorotondo (274 Bq/m^3^). Among the municipalities of Lecce province, the mean radon concentration found in these 20 municipalities was between 66 and 310 Bq/m^3^. The highest average concentrations were evident in Minervino (310 Bq/m^3^), Campi Salentina (266 Bq/m^3^) and Copertino (235 Bq/m^3^) (Figure 4).

The characteristics of the soils of the sample buildings showed that the most widespread types of soil in the examined municipalities are of organogenic and debris limestone; local sandstones and those of the miocene–pleistocene age were inferior to sandstone from the oligocene. Regarding the year of construction, the highest average number of buildings was observed in the decade 1971–1980, and four of the five municipalities previously mentioned (excluding the town of Locorotondo, where the largest number of buildings is attributable to the period before 1919) were verified to be built in the years 1961–1980. Regarding the concentration of the road level, the highest percentage of all the constructions measured have only one floor on the road (39%). The highest percentages of buildings with a single floor are present in the municipalities with the highest radon concentrations: Campi Salentina (64%); Copertino (61%); Minervino (56%); and Locorotondo (59%). The municipality of Gravina di Puglia has the largest percentage of buildings with three floors (39%). The most used construction material is the masonry bracket (57%), followed by reinforced concrete (33%) and other materials (10%). Among the five municipalities with the highest average radon concentration, four have the highest percentages of masonry bearing (Campi Salentina (68%), Copertino (71%), Locorotondo (58%), and Minervino (96%)), while the municipality of Gravina di Puglia shows one of the highest percentage values of reinforced concrete (46%). The number of the most frequently represented interiors in the considered buildings is one internal (56%) building, followed by two extensions (20%) and three or four interior (11%) buildings, with more interiors being represented in very low percentages. Among the five municipalities to which we referred previously, three have only one interior (Campi Salentino (68%), Minervino (96%), and Locorotondo (58%)), while the other two have measures of an internal number more than nine (Gravina di Puglia (75%) and Copertino (46%)).

### 3.2. Univariate Analysis

Because the stochastic effects of radon follow the non-threshold theory, the risks of observing radon values above the 75th percentile of the cumulative distribution of the mean concentrations detected (145 Bq/m^3^) were analyzed; this value represents a value below the limit imposed by regional laws and helps us to understand the role of some determinants. The risk levels odds ratio (ORs) were observed, although they were not statistically significant given the low power of the study and can be used in an indicative and non-causal way. For the year of construction, the highest risks were highlighted for the years 1971–1980 (OR = 1.77, 0.41–8.17), 1981–1990 (OR = 1.16, 0.27–5.06) and 1991–2000 (OR = 2.77, 0.62–14.03). The number of rooms in houses most at risk of producing a high radon level was one room (OR = 1.77, 0.41–8.17). The type of masonry represented was load-bearing masonry (OR = 2.77, 0.62–14.3). The soil types were represented by pelites, clayey sands, gravels or conglomerates, calcarenites, and permeable lipotypes (OR = 2.41, 0.51–10.64). Regarding the risk related to the number of floors above the road, the high risk was linked to the first floor (OR = 4.58, 0.94–29.21) (Table 1).

### 3.3. Multivariate Analysis

The objectives of the study were to evaluate the interdependence or independence of all these variables using the unconditional logistic regression model and check that the adjusted risk of observing radon values above the 75th percentile (145 Bq/m^3^) was represented fundamentally and significantly by the time of construction in 1991–2000 (OR = 4.94, 1.02–23.81). The major influences are related to the number of upper floors (1) and type of load-bearing masonry, while the other factors do not seem to contribute to these concentrations (Table 2).

### 3.4. Calculation of the Estimates of Lung Cancer Attributable to Radon

The determination of estimates (number of neoplasms per 10 × 10^3^ inhabitants). The method previously described shows that the highest frequencies were present in the counties of Campi Salentina (3.34/10,000 inhabitants), Copertino (2.95/10,000 inhabitants), Gravina di Puglia (5.36/10,000 inhabitants), Locorotondo (3.44/10,000 inhabitants), Minervino (3.89/10,000 inhabitants), and Otranto (2.49/10,000 inhabitants). The number of tumors attributable to radon in the provinces of Bari and Lecce and in Apulia were lower (province of Bari: 1.17/10,000 inhabitants; province of Lecce: 1.85/10,000 inhabitants; Apulia: 1.43/10,000 inhabitants). It was also possible to estimate the distribution of radon decay products (^210^Po, ^214^Pb, ^214^Bi) in the individual countries and cities in the study starting from the average annual concentration, and it was observed that the highest values were found in the municipalities with high average annual concentrations and a ^214^Bi presence higher than that of the other isotopes (Table 3).

## 4. Discussion

The data of the 51 surveys of radon in buildings, distributed between the provinces of Bari and Lecce, showed an average between the two provinces of 114.20 Bq/m^3^, which is higher than the regional average of 60 Bq/m^3^ and the national average of 70 Bq/m^3^.

As expected, there were wide variations in the average concentration (between a minimum of 18 Bq/m^3^ for Valenzano and a maximum of 427 Bq/m^3^ for Gravina di Puglia). In most samples, the average results obtained did not reveal any alarming values for health effects compared with the values of the Recommendation of the European Directive 2013/59/Euratom (<300 Bq/m^3^). However, in some cases, the average values of radon were found to be considerably high (Gravina di Puglia: 427 Bq/m^3^; Minervino: 310 Bq/m^3^; Locorotondo: 274 Bq/m^3^; Copertino: 235 Bq/m^3^) to be a concern due to recent studies demonstrating the correlation between high levels of radon and lung carcinoma. These differences in the concentrations of the radioactive gas are due to several factors, both urban-constructive and geographical. Among the parameters used as study variables, those that most affected the high levels of radon were therefore selected as risk factors (higher than the 75th percentile), they are as follows: buildings constructed between 1991 and 2000, buildings with one floor above the street level, buildings with the presence of one interior, and buildings constructed of load-bearing masonry or other material (e.g., tuff). In these structural characteristics, the root causes of radon levels can likely be traced because, in recent years, following the energy crisis, there has been a diversification of building techniques and housing practices, leading to a hypersigillation of the houses and a consequential accumulation of the concentration of gas inside. Moreover, it is evident that buildings of the simplest architectural construction (such as those with one room or with only one floor above the street level) have higher levels of radon because they are made, in most cases, with poorer materials or with a higher level of radioactivity such as the supporting stone or tuff. Additionally, the geographic placement factor of the houses affected the different radon values due to the presence or absence of seismic faults or tectonic contacts, which could favor the escape of gas from the subsoil through the cracks, both concerning the composition and type of soil. In fact, the soil formed by pebbles, clayey sands, gravels or conglomerates, calcarenites, and permeable lithotypes favors the escape of radon from underground to indoor spaces due to greater porosity and permeability. While protective properties have been found in houses built in the early 1900s, this not so for houses with an interior number of more than one room, those of average size with several rooms between three and eight, and those built at a soil formed by limestone, dolomitic limestone, and dolomites locally with poorly permeable marly levels made of clay.

The epidemiological data listed in Table 3 provide an estimate of the lung cancer risk of 51 Apulian municipalities scattered between the provinces of Bari and Lecce. The method used to estimate the lung cancer risk was similar to the method used to evaluate miners, and, although this analogy cannot be considered perfect as a result of the different characteristics and variables of study, their potential risks of lung carcinoma may be due, in part, to the risks of cancer related to housing. Housing characteristics, such as floors, sump holes, ventilation, and heating systems were suspected for high indoor radon levels and health consequences [21]. A recent survey on radon levels in indoor environments of the university hospital in Bari reported an average concentration of radon lower than the WHO reference level (100 Bq/m^3^) [22]. A radon survey conducted in 2013–2014 in various dwellings of Peninsula Salentina of the Apulia region, in Southern Italy, found that higher radon concentrations were found in bedrooms than in living rooms, underground, and at ground level rather than upper floors. Finally, the median radon concentration was significantly higher for older houses than for those built after 1960, probably due to the lower ventilation rate in older houses because they have smaller or fewer windows, greater structural deterioration, and poor ground insulation [23]. In opposition to these results, a survey of household radon gas levels and risk factors in Southern Alberta found that homes built in 1992 or later had radon levels higher than older homes; the reasons could be the greater insulation practices of recent buildings, the recent increases in home floor-plan sizes in Alberta, and recently increased building height thresholds that generate negative pressure at the basement level, all of which cause more indoor radon [24].

The average WLM per year values of the Apulian studied municipalities was 0.41 for the province of Bari, 0.65 for the province of Lecce, and 0.51 for the average of the two provinces. Therefore, only the average WLM value of the countries of the province of Bari was in line with the Italian average in the domestic environment at 0.41 WLM per year and corresponded to a risk of developing lung cancer related to radon equal to 11.66 × 10^5^, that is 1.2 cases/10,000 inhabitants. On the other hand, it is more worrying for the average data of Lecce to be 0.51 WLM, which is estimated to lead to a risk of developing lung cancer in the Lecce area of almost 1.9 cases/10,000 inhabitants. Moreover, the estimated crude incidence rate of lung cancer of the year 2015, measured based on the cancer registry of Puglia, incidence, mortality and survival of oncological diseases in Puglia indicated a higher incidence of such tumors in the province of Lecce than in the other areas, especially in the male population [25].

The cities with the highest risk of lung cancer development are Campi Salentina and Minervino for Lecce province with 1.18 WLM and 1.38 WLM, respectively, corresponding to 3.34 and 3.89 cancer cases/10,000 inhabitants. Among the cities of the Bari province, most of those with an increased risk of exposure to radon were in Locorotondo (1.22 WLM) and Gravina di Puglia (1.89 WLM) with 3.44 and 5.36 tumors/10,000 inhabitants, respectively.

Certainly, the study presents the limits of an observational study regarding the current data. Because of the small number of observations and lack of analytical data—for example, on the ventilation and air change rate for each building test and type of flooring [22]. Thus, these estimates need further validation to avoid bias.

However, it should be noted that despite some countries presenting alarming results on the probability of lung cancer from indoor radon, these results cannot be considered accurate because of the reduced number of samples and measures available for the study and because the values are influenced by the normal incidence of variable lung cancer with sex (higher in men) and with some lifestyle habits (cigarette smoking in particular).

Nevertheless, the results obtained can be used to estimate the risk and develop some points of reflection on the radon indoor issue and then, in the future, develop a broader, complete and effective experimental epidemiological study on this topic. A review underlined the need to consider smoking and other factors in the research on the correlation between radon exposure and lung cancer incidence [26]. Another review showed that indoor radon is the second highest risk factor of lung cancer after cigarette smoking among ever-smokers and the first among non-smokers; however, few studies have considered important factors such as smoking and the age of a building in the assessment of the association between exposure to residential radon and lung cancer risk [27]. The low incidence of lung cancer associated with radon exposure in the general population, as well as the strong carcinogenic effects of smoking and other environmental materials make the assessment of the real risk of indoor radon on lung cancer difficult. Therefore, studies in never-smokers are very important. However, studies including both smokers and never-smokers are also necessary to evaluate the interactions between radon and smoking for smoking-related lung cancer [28].

The development of the arms and nuclear energy industries after the Second World War meant that many countries began mining uranium, with the result that many workers were exposed to radon. Even workers in other types of mines (iron mines in Sweden or tin in the Republic of China) were largely exposed to radon when they were operating in geological strata rich in uranium. Numerous studies have been carried out on the health status of these workers, considering their exposure to radon for several decades. Convergent conclusions have been reached: radon undoubtedly increases the risk of lung cancer in miners [4]. Hoffmann and colleagues found that a retrospective assessment of radon exposure for French uranium miners could explain the association between occupational radon exposure and lung cancer mortality [29].

Furthermore, by measuring the exposure levels reached by these workers, it was possible to estimate to what extent the risk of lung cancer increases with exposure. Here, too, the various studies arrived at very similar results.

Studies on miners explicitly cover a particular segment of the population, adults who have been exposed for relatively short periods (e.g., 40 h per week) and for a limited number of years. In addition, miners are exposed to other factors that may play a role in the incidence of lung cancer because they, by definition, work in an atmosphere loaded with powders. Furthermore, the miners studied were frequent smokers, and the risk of lung cancer related to smoking is well known [26,27].

In contrast, members of the population (both sexes and all ages) are continually exposed to radon. On average, they smoke less and breathe purer air.

These differences justify the study of risks related to the presence of radon in homes [13]. In fact, the degree and urgency of the preventive measures to be taken depend on the magnitude of the risk that a given level of exposure implies (considering age, sex and other factors). These studies are ongoing in many countries. They are particularly complex, sometimes explaining the contradictory results. In a few years, there will be several new results offering a solution to the remaining unresolved issues.

It has been widely demonstrated that tobacco smoking is responsible for most lung cancers in both men and women. Studies on the combined effects of exposure to radon and cigarette smoke show that the total effect of such exposures is much greater than the sum of the two effects. In other words, smoking increases the risk of lung cancer related to radon considerably and vice versa. In addition, radon alone is the second leading cause of lung cancer after tobacco. In particular, the concomitant effect of the two factors increases the risk of developing lung cancer by approximately 15 times [30].

A combined analysis of these studies [31,32,33], especially analysis of European studies [34,35], allowed the risk quantification. This quantification presents a linear relationship of risk with exposure, without a detectable threshold (stochastic effect), and is quantifiable in an increase of approximately 16% per 100 Bq/m^3^ in the indoor radon concentration (excess of relative risk equal to 0.16, with a confidence interval 0.05–0.31) [36].

In a closed environment, a mixture of radon and its descendants is never balanced in the air. To evaluate the dose derived from staying in an environment in which radon is present, the respective concentration in free form and in aerosols must be known for each of the radioisotopes (different methods of deposition in the respiratory system) [37].

In a cohort study, no statistically significant association was observed between residential radon exposure and lung cancer; however, after restricting the analysis to a sample of greater median age, the risk of lung cancer would have been higher. This is probably due to a consequent and greater follow-up time, which is necessary to induce lung cancer. [38]. A study conducted in Alberta in 2011 estimated the population’s attributable risk of lung cancer due to radon exposure: it was higher among those who had never smoked than among ever-smokers. However, since only approximately 10% of lung cancer cases occur in nonsmokers, the estimated total number of excess cases was higher for ever smokers than for never smokers [39].

Torres-Duran and colleagues also found that residential radon is the second highest risk factor for lung cancer in smokers and the most important risk in never-smokers. They also found that several studies described the synergy between exposure to residential radon and tobacco smoke. Therefore, they underlined that if we do not consider residential radon when calculating the lung cancer risk, we are misclassifying the probabilities of lung cancer development [40].

Other factors included in the risk prediction models (personal history of respiratory disease, such as chronic obstructive pulmonary disease; family history of lung cancer; race; body mass index) have not shown a strong association with lung cancer risk compared with radon. Torres-Duran and colleagues showed that a more accurate assessment of risk for lung cancer from radon exposure may be ascertained if a person has lived in the same residence for many years (at least 10–15 years) [40]. A study about cancer mortality in Galicia, Spain, showed that the radon concentration in the interior of homes in Galicia is statistically associated with higher lung, stomach and brain cancer mortality among women [41].

A radon-prone area in Spain was analyzed, and it was found that the indoor radon concentration was generally greater than the contribution from soil exhalation, especially in new dwellings (1980–2014). Its concentration in dwellings built in the traditional style (1729–1940) was significantly lower than that in new houses. This difference may be a result of the air tightness of the dwellings [42].

In Utah, nonmetropolitan counties have a higher lung cancer incidence than metropolitan counties. Exposure to high levels of radon explain the disparity in lung cancer incidence rates among nonmetropolitan Utah counties [43].

Radon contributes to 12% of all cases of lung cancer annually in Norwegian homes. Therefore, a reduction of the indoor radon concentration may be the most important measure to reduce the risk of lung cancer, especially for smokers who do not want to or cannot quit [44].

## 5. Conclusions

This work is an attempt to study and analyze how some factors can influence or affect the concentration of radon in a closed living environment through original software created for this purpose. Thus, it was an observational study that tended to demonstrate the possible effects of various risk or protective factors, in this case, radon levels. This study analyzed the data that were available without any intervention by the researchers.

In any case, the work presents limits and problems related to the small size of the database and the low analytical description of house characteristics available on current Arpa data.

The danger of radon gas has been known since ancient times. In fact, it was mentioned by Lucretius in the “De Rerum Natura” (book VI) as an unknown cause of deadly diseases for a significant group of miners. However, only in the last few years has there been greater awareness of the impact on human health, highlighting the need to tackle this problem more rigorously and carry out numerous epidemiological studies, such as this study, to have greater awareness and knowledge on the subject. Additionally, the results on the probabilistic estimate of cancer risk due to radon are worrying for some of the analyzed Apulian municipalities; so much so, that they can be said to be the cause of 3–5 lung carcinomas a year against 10 × 10^3^ inhabitants (as in Gravina di Puglia). As recently stated, it would be appropriate to use the precautionary principle implying the need to limit, as much as possible, the exposure to indoor radon. This principle is based on the debated hypothesis of the absence of a threshold in the dose-response relationship between exposure to radon and oncogenic effects. The statistical study here developed can be used in areas with a high concentration of radon for the adoption and application of targeted remedial and mitigation actions and to provide useful suggestions to draft building plans, especially in heavily populated areas.

## Figures and Tables

**Table 1:** Risk (ORs) distribution to observe Radon high mean concentrations (>145 Bq/m^3^) by multiple determinant factors.

	Variables Labels		Radon Mean Concentration (>145 [Bq/m^3^])			OR	LCI (95%)	UCI (95%)
			Yes	No.	Total			
YEAR OF CONSTRUCTION	<1919	Yes	9	16	25			
		Yes	4	22	26	0.32	0.06	1.44
	1919-1945	No	7	18	25			
		Yes	6	20	26	0.77	0.17	3.27
	1946-1960	No	6	19	25			
		Yes	7	19	26	1.16	0.27	5.06
	1961-1970	No	6	19	25			
		Yes	7	19	26	1.16	0.27	5.06
	1971-1980	No	5	20	25			
		Yes	8	18	26	1.77	0.41	8.17
	1981-1990	No	6	19	25			
		Yes	7	19	26	1.16	0.27	5.06
	1991-2000	No	4	21	25			
		Yes	9	17	26	2.77	0.62	14.03
	2001-2005	No	6	19	25			
		Yes	7	19	26	1.16	0.27	5.06
	> 2005	No	5	20	25			
		Yes	8	18	26	1.77	0.41	8.17
			**Yes**	**No.**	**Total**			
NUMBER OF ROOMS	1	No	5	20	25			
		Yes	8	18	26	1.77	0.41	8.17
	2	No	7	18	25			
		Yes	6	20	26	0.77	0.17	3.27
	3 April	No	8	17	25			
		Yes	5	21	26	0.5	0.11	2.16
	5 August	No	8	17	25			
		Yes	5	21	26	0.5	0.11	2.16
	15 September	No	6	18	24			
		Yes	7	20	27	1.05	0.24	4.56
	>15	No	6	18	24			
		Yes	7	20	27	1.05	0.24	4.56
			**Yes**	**No.**	**Total**			
NUMBER OF FLOOR	First	No	16	22	38			
		Yes	10	16	26	4.58	0.94	29.21
	Second	No	8	17	25			
		Yes	5	21	26	0.5	0.11	2.16
	Third	No	9	16	25			
		Yes	4	22	26	0.32	0.06	1.44
	Fourth	No	8	17	25			
		Yes	5	21	26	0.5	0.11	2.16
			**Yes**	**No.**	**Total**			
WALL TYPES	Supporting masonry	No	4	21	25			
		Yes	9	17	26	2.77	0.62	14.3
	Reinforced concrete	No	10	15	25			
		Yes	3	23	26	0.19	0.03	0.95
	Other	No	4	21	25			
		Yes	9	17	26	2.77	0.62	14.3
			**Yes**	**No.**	**Total**			
* SOIL TYPES	1	No	7	28	35			
		Yes	6	10	16	2.41	0.51	10.64
	2	No	10	29	39			
		Yes	3	9	12	0.96	0.14	5
	3	No	8	22	30			
		Yes	5	16	21	0.85	0.18	3.68
	4	No	12	25	37			
		Yes	1	13	14	0.16	0.003	1.35

* Soil types. (1) Pelts, clayey sands, gravel or conglomerates, calcarenites, permeable lithotypes; (2) Calcues, dolomite limestones and dolomites; locally at the bottom bauxite levels; (3) Organic and detritic calves; locally sandy; (4) Calcues, dolomite limestones, and dolomites locally with low permeable marzipan levels made up of clay. Low confidence interval (LCI); Upper confidence interval (UCI).

**Table 2:** Adjusted risk (ORs) to observe Radon concentrations above the 75th percentile (145 Bq/m^3^).

			Number of Obs.	51.00		
			(likelihood Ratio) χ^2^ (5)	11.24		
			Prob > χ^2^	0.04		
			(Determination Coefficinet) Pseudo R^2^	0.19		
**Radon**	**Odds Ratio**	**Std. Err.**	**Z (Standardized Deviation)**	**p (Z)**	**LCI (95%)**	**UCI (95%)**
Construction year 1991–2000	4.95	3.97	1.99	0.05	1.03	23.81
Building with 1 room	0.33	0.46	−0.79	0.43	0.02	5.13
Building at first floor	6.23	8.50	1.34	0.18	0.43	90.37
Supporting masonry	2.98	2.55	1.27	0.20	0.56	15.98
Pelts, clayey sands, gravel or conglomerates, calcarenites, permeable lithotypes	0.23	0	−0.80	0.42	0.31	2.4

**Table 3:** Distributions of radon concentrations and its decay isotopes and the expected annual frequency of radon attributable lung cancer by city.

Municipalities	Annual Mean Concentration	f	Annual Hours	Annual Cumulative Exposure	EEC	^210^ Po (CA)	^214^ Pb (CB)	^214^ Bi (Cc)	EEC of 1 WLM	WLM	Probability of Cancer	Expected Annual Cancer Number (/10000)
**Province of Bari**												
Acquaviva delle Fonti	68	0.5	5590	3.8	1.9	0.49	0.49	4.2	6.3	0.3	2.83	0.85
Adelfia	57	0.5	5590	3.2	1.59	0.41	0.41	3.5	6.3	0.25	2.83	0.72
Andria	88	0.5	5590	4.9	2.46	0.64	0.64	5.4	6.3	0.39	2.83	1.1
Bari	96	0.5	5590	5.4	2.68	0.7	0.7	5.9	6.3	0.43	2.83	1.21
Barletta	77	0.5	5590	4.3	2.15	0.56	0.56	4.8	6.3	0.34	2.83	0.97
Bisceglie	161	0.5	5590	9	4.5	1.17	1.17	9.9	6.3	0.71	2.83	2.02
Bitonto	43	0.5	5590	2.4	1.2	0.31	0.31	2.7	6.3	0.19	2.83	0.54
Bitritto	35	0.5	5590	2	0.98	0.25	0.25	2.2	6.3	0.16	2.83	0.44
Capurso	115	0.5	5590	6.4	3.21	0.84	0.84	7.1	6.3	0.51	2.83	1.44
Casamassima	89	0.5	5590	5	2.49	0.65	0.65	5.5	6.3	0.39	2.83	1.12
Cassano delle Murge	85	0.5	5590	4.8	2.38	0.62	0.62	5.3	6.3	0.38	2.83	1.07
Castellana Grotte	48	0.5	5590	2.7	1.34	0.35	0.35	3	6.3	0.21	2.83	0.6
Corato	65	0.5	5590	3.6	1.82	0.47	0.47	4	6.3	0.29	2.83	0.82
Gioia del Colle	145	0.5	5590	8.1	4.05	1.05	1.05	9	6.3	0.64	2.83	1.82
Giovinazzo	76	0.5	5590	4.2	2.12	0.55	0.55	4.7	6.3	0.34	2.83	0.95
Gravina in Puglia	427	0.5	5590	23.9	11.93	3.1	3.1	26.4	6.3	1.89	2.83	5.36
Locorotondo	274	0.5	5590	15.3	7.66	1.99	1.99	16.9	6.3	1.22	2.83	3.44
Modugno	47	0.5	5590	2.6	1.31	0.34	0.34	2.9	6.3	0.21	2.83	0.59
Mola di Bari	20	0.5	5590	1.1	0.56	0.15	0.15	1.2	6.3	0.09	2.83	0.25
Molfetta	59	0.5	5590	3.3	1.65	0.43	0.43	3.6	6.3	0.26	2.83	0.74
Monopoli	78	0.5	5590	4.4	2.18	0.57	0.57	4.8	6.3	0.35	2.83	0.98
Noci	32	0.5	5590	1.8	0.89	0.23	0.23	2	6.3	0.14	2.83	0.4
Noicattaro	122	0.5	5590	6.8	3.41	0.89	0.89	7.5	6.3	0.54	2.83	1.53
Polignano a Mare	95	0.5	5590	5.3	2.66	0.69	0.69	5.9	6.3	0.42	2.83	1.19
Putignano	68	0.5	5590	3.8	1.9	0.49	0.49	4.2	6.3	0.3	2.83	0.85
Rutigliano	43	0.5	5590	2.4	1.2	0.31	0.31	2.7	6.3	0.19	2.83	0.54
Ruvo di Puglia	145	0.5	5590	8.1	4.05	1.05	1.05	9	6.3	0.64	2.83	1.82
Toritto	37	0.5	5590	2.1	1.03	0.27	0.27	2.3	6.3	0.16	2.83	0.46
Trani	116	0.5	5590	6.5	3.24	0.84	0.84	7.2	6.3	0.51	2.83	1.46
Triggiano	65	0.5	5590	3.6	1.82	0.47	0.47	4	6.3	0.29	2.83	0.82
Valenzano	18	0.5	5590	1	0.5	0.13	0.13	1.1	6.3	0.08	2.83	0.23
**Province of Lecce**												
Campi Salentina	266	0.5	5590	14.9	7.43	1.93	1.93	16.4	6.3	1.18	2.83	3.34
Castrignano del capo	157	0.5	5590	8.8	4.39	1.14	1.14	9.7	6.3	0.7	2.83	1.97
Copertino	235	0.5	5590	13.1	6.57	1.71	1.71	14.5	6.3	1.04	2.83	2.95
Cutrofiano	104	0.5	5590	5.8	2.91	0.76	0.76	6.4	6.3	0.46	2.83	1.31
Galatina	120	0.5	5590	6.7	3.35	0.87	0.87	7.4	6.3	0.53	2.83	1.51
Leverano	77	0.5	5590	4.3	2.15	0.56	0.56	4.8	6.3	0.34	2.83	0.97
Maglie	173	0.5	5590	9.7	4.84	1.26	1.26	10.7	6.3	0.77	2.83	2.17
Melendugno	98	0.5	5590	5.5	2.74	0.71	0.71	6.1	6.3	0.43	2.83	1.23
Minervino	310	0.5	5590	17.3	8.66	2.25	2.25	19.1	6.3	1.38	2.83	3.89
Nardò	125	0.5	5590	7	3.49	0.91	0.91	7.7	6.3	0.55	2.83	1.57
Otranto	198	0.5	5590	11.1	5.53	1.44	1.44	12.2	6.3	0.88	2.83	2.49
Poggiardo	126	0.5	5590	7	3.52	0.92	0.92	7.8	6.3	0.56	2.83	1.58
Ruffano	90	0.5	5590	5	2.52	0.65	0.65	5.6	6.3	0.4	2.83	1.13
Rutigliano	43	0.5	5590	2.4	1.2	0.31	0.31	2.7	6.3	0.19	2.83	0.54
Salve	128	0.5	5590	7.2	3.58	0.93	0.93	7.9	6.3	0.57	2.83	1.61
Squinzano	66	0.5	5590	3.7	1.84	0.48	0.48	4.1	6.3	0.29	2.83	0.83
Supersano	88	0.5	5590	4.9	2.46	0.64	0.64	5.4	6.3	0.39	2.83	1.1
Surbo	184	0.5	5590	10.3	5.14	1.34	1.34	11.4	6.3	0.82	2.83	2.31
Taviano	117	0.5	5590	6.5	3.27	0.85	0.85	7.2	6.3	0.52	2.83	1.47
Tricase	78	0.5	5590	4.4	2.18	0.57	0.57	4.8	6.3	0.35	2.83	0.98
